# Thermal Stability of Fructooligosaccharides Extracted from Defatted Rice Bran: A Kinetic Study Using Liquid Chromatography-Tandem Mass Spectrometry

**DOI:** 10.3390/foods11142054

**Published:** 2022-07-11

**Authors:** Hoang Phuong Le, Diep Thanh Nghi Hong, Thi Thao Loan Nguyen, Thi My Hanh Le, Shige Koseki, Thanh Binh Ho, Binh Ly-Nguyen

**Affiliations:** 1 Department of Food Technology, Can Tho University, Can Tho 900000, Vietnam; lhphuong@vnkgu.edu.vn (H.P.L.); hdtn.ct@gmail.com (D.T.N.H.); 2 Faculty of Food Sciences and Health, Kien Giang University, Rach Gia 920000, Vietnam; 3 Ho Chi Minh City Industry and Trade College, Ho Chi Minh City 700000, Vietnam; thloankt@yahoo.com; 4 Faculty of Tourism, University of Finance—Marketing, Ho Chi Minh City 700000, Vietnam; hanhvn1@gmail.com; 5 Research Faculty of Agriculture, Hokkaido University, Sapporo 060-8589, Japan; koseki@bpe.agr.hokudai.ac.jp; 6 Faculty of Agriculture and Natural Resources, An Giang University, Vietnam National University Ho Chi Minh City, Long Xuyen 90116, Vietnam; htbinh@agu.edu.vn

**Keywords:** 1-fructosyl-nystose, 1-kestose, nystose, fructooligosaccharides, FOS, rice bran, UPLC-ESI-MS/MS, thermal degradation kinetics

## Abstract

Thermal degradation kinetics of fructooligosaccharides (FOS) in defatted rice bran were studied at temperatures of 90, 100, and 110 °C. FOS extracted from rice bran and dissolved in buffers at pH values of 5.0, 6.0, and 7.0 were prepared for the thermal treatments. The residual FOS (including 1-kestose (GF2), nystose (GF3), and 1F-fructofuranosylnystose (GF4)) contents were determined using the ultra-performance liquid chromatography-electrospray ionization-tandem mass spectrometry (UPLC-ESI-MS/MS) method. The results showed that the thermal degradation kinetics of GF2, GF3, and GF4 followed a first-order kinetic model. Thermal degradation rate constants (*k* values) of GF2, GF3, and GF4 at different temperature and pH values were estimated using the first-order kinetic equation and SAS 9.1. As a result, these *k* values decreased gradually as the pH of the sample increased from 5.0 to 7.0. The Arrhenius model was applied to describe the heat dependence of the *k*-values. The activation energy (*E_a_*) was calculated for each case of GF2, GF3, and GF4 degradation at pH values of 5.0, 6.0, and 7.0. The result showed that rice bran FOS is very thermostable at neutral pH while more labile at acidic pH.

## 1. Introduction

‘Fructooligosaccharides’ (FOS) is the common name for fructose oligomers, including three major representatives known as 1-kestose (GF2), nystose (GF3), and 1F-fructofuranosylnystose (GF4). FOS are widely present in a wide variety of foods and feedstuffs. They are naturally occurring sugars with potentially beneficial nutritional effects [1,2]. FOS are not enzymatically digested and absorbed in the upper digestive tract, reaching the colon intact before experiencing microbial fermentation. FOS selectively stimulates the reproduction of bifidobacteria, a group of beneficial bacteria naturally found in the human colon [3,4,5]. Short-chain fatty acids (SCFAs), derived from FOS fermentation by the intestinal microbiota, can favor the growth of health-promoting bacteria, including *Bifidobacterium* spp. and *Lactobacillus* spp., while reducing or maintaining pathogenic populations (e.g., *Clostridium* spp. and *Escherichia coli*) at low levels [6,7,8]. Thus, FOS, as small soluble dietary fibers, exhibit prebiotic activity [9,10,11,12,13,14,15,16]. In addition, there has been growing evidence supporting the hypothesis that SCFAs exert crucial physiological effects on several organs, including the brain [17,18,19]. This idea is supported by studies in animals and humans showing that gut microbiota dysbiosis has been implicated in behavioral and neurologic pathologies, such as depression, Alzheimer’s and Parkinson’s diseases, and autism spectrum disorder [19,20,21,22,23]. Microbiota manipulation and SCFA administration have been proposed as treatment targets for such diseases [19,24,25].

Rice (*Oryza sativa*) is a global crop that has a long history of safe usage as an indispensable food for humans [26]. The use of rice and its co-products (including oil, bran, husk, straw, etc.) in functional foods is not a novelty, of which a diverse number of bioactive compounds have been identified as fructooligosaccharides, ferulic, γ-oryzanol, etc. Among those, more and more fructooligosaccharides are concerned to be incorporated in many food applications with thermal treatments extensively applied among other conventional processes. In this context, the processed stability of these compounds should be essentially evaluated. Thus far, the acid hydrolysis kinetics of five commercially available mixes of oligofructose samples (Actilight 950P, Raftilose P95, Fibrulose 97, Fibruline instant, and Fibruline Long Chain) incubated in an acidic media were reported by Blecker et al. [27]. L’homme et al. [2] studied the heat and pH hydrolysis kinetics of standard FOS (Wako, Neuss, Germany). Courtin et al. [28] reported findings of the heat and pH stability of prebiotic non-digestible wheat bran-derived arabinoxylooligosaccharides, xylooligosaccharides, and chicory root inulin-derived FOS. However, the data on the degradation kinetics of GF2, GF3, and GF4 crude extracts obtained from rice bran have not been documented yet.

The aim of the present study was to investigate the temperature-pH degradation kinetics of FOS extracted from rice bran in order to gain insights into the effects of thermal processing on the FOS content of food products.

## 2. Materials and Methods

### 2.1. Materials

Defatted rice bran was provided by Wilmar Agro Vietnam (Can Tho, Vietnam). Cellulase preparation was provided by Novozymes (Bagsvaerd, Denmark) and delivered by Trung Son Technology (Ho Chi Minh City, Vietnam). The cellulase preparation is in brown color, noted with an activity of 700 EGU/g and the best-storing temperature of 4 °C. Standard FOS set, including GF2, GF3, and GF4, with a purity 99% was purchased from Fujifilm Wako Pure Chemical Corporation (Osaka, Japan). Acetonitril, water, and methanol for UPLC analysis were from Merck (Darmstadt, Germany).

### 2.2. Extraction of FOS from Rice Bran

The extraction of FOS from defatted rice bran was performed according to the method of Patindol et al. [29] with minor modification. Ten grams of defatted rice bran were dispersed in 100 mL of deionized water and heated at 100 °C for 30 min using magnetic stirrer. The mixture was then allowed to cool down to room temperature, being blended for 2 min at 5000 rpm using the IKA T25 disperser (IKA T25 digital Ultra-Turrax^®^, Merck, Darmstadt, Germany), being added with 0.1 mL cellulase, and incubated in a water bath shaker at 50 °C for 1 h. The mixture was then blended for 2 min using the IKA T25 disperser (5000 rpm), added with an equal volume of ethanol, stirred, allowed to stand for 15 min, and centrifuged at 5252× *g* for 12 min (Rotixa 500RS centrifuge, Hettich, Tuttlingen, Germany). The supernatant was recovered and dried by lyophilization. The dried powder was stored at −20 °C for further use. The yield of fructooligosaccharides extracted from rice bran was about 0.7 g/kg rice bran.

### 2.3. Thermal Degradation of Rice Bran-Extracted GF2, GF3, and GF4

Rice bran crude extracts (100 mg) and buffers (pH 5.0, 6.0, or 7.0; 50 mL) were added to Falcon 50 mL conical centrifuge tubes (Fisher Scientific, Waltham, MA, USA) for well mixing. The mixed samples of 5 mL were enclosed in Kimax^®^ culture tubes 16 × 100 mm with closed cap (DWK Life Sciences, Milville, NJ, USA). Isothermal treatments were conducted in a temperature-controlled block heater (MG-2200, Tokyo Rikakikai (EYELA), Tokyo, Japan). After isothemal treatment, the samples were immediately cooled down in ice water and measured for the residual contents of GF2, GF3, and GF4.

### 2.4. Analysis of FOS by UPLC-ESI-MS/MS

The rice bran-extracted samples thermally treated were analyzed for the residual GF2, GF3, and GF4 contents using an ultra-performance liquid chromatography system equipped with an ESI (Waters Corporation, Milford, MA, USA) coupled to an MS/MS system (Xevo TQS Micro, (Waters Corporation, Milford, MA, USA). The chromatographic separation was performed on a Luna amino—NH_2_ column (150 mm × 2 mm × 5 µm) (Phenomenex, Torrance, Los Angeles, CA, USA) [30]. The elution was performed at a constant flow rate of 0.45 mL/min following the program presented in Table 1, with an injection volume of 10 µL and an overall run time of 5 min per sample injection. The Xevo TQS Micro MS/MS system runs in negative ionization mode. The initial optimization parameters are as follows: ionization potential (capillary) of 2.5 kV, source temperature of 150 °C, desolvation temperature of 500 °C, and nitrogen gas rate of 800 mL/h. The fragmentation conditions for measurement of GF2, GF3, and GF4 are also reported in Table 1.

### 2.5. Kinetic Data Analysis

Degradation of GF2, GF3, and GF4 can be described by a first-order kinetic model [2] (Equation (1)):

(1) $$ln\left(\frac{A}{A_{0}}\right)=-kt,$$

where *A*_0_ and *A* are respectively initial- and remaining concentrations at time *t* (min); and *k* is the degradation rate constant (min^−1^).

Equation (1) is valid under isothermal conditions, whereby the degradation rate constant *k* can be determined from a linear regression analysis of *ln*(*A*/*A*_0_) versus time.

The temperature dependence of the degradation rate constants can be estimated using the Arrhenius equation (Equation (2)):

(2) $$ln\left(k\right)=ln\left(k_{0}\right)+\left[\frac{E_{a}}{R_{T}}\left(\frac{1}{T_{0}}-\frac{1}{T}\right)\right],$$

where *T* is absolute temperature (K); *T*_0_ is reference absolute temperature (K); *k*_0_ is *k* at *T*_0_ (min^−1^); *E_a_* is activation energy (kJ mol^−1^), and *R_T_* (8.314 J mol^−1^ K^−1^) is the universal gas constant.

The activation energy can be estimated by linear regression analysis of the natural logarithm of the rate constant versus the inverse of absolute temperature.

Many empirical polynomial models describing the relationship between the predicted response (i.e., *k* value in the present case) and the independent variables (i.e., temperature and pH) have been formulated [31,32,33,34,35]. Among those, the second-order polynomial model for two factors can be addressed in this study (Equation (3)).

(3) $$Y=\beta_{0}+\beta_{1}X_{1}+\beta_{2}X_{2}+\beta_{11}X_{1}^{2}+\beta_{22}X_{2}^{2}+\beta_{12}X_{1}X_{2},$$

where *Y* is predicted response; *β*_0_ is constant; *β*_1_, *β*_2_, *β*_11_, *β*_22_, and *β*_12_ are unknown parameters of variables for linear, quadratic, and interaction terms, respectively; *X*_1_ and *X*_2_ are independent variables.

## 3. Results and Discussion

### 3.1. Development of Standard Curves for GF2, GF3, and GF4 Analysis

**Figure 1:**
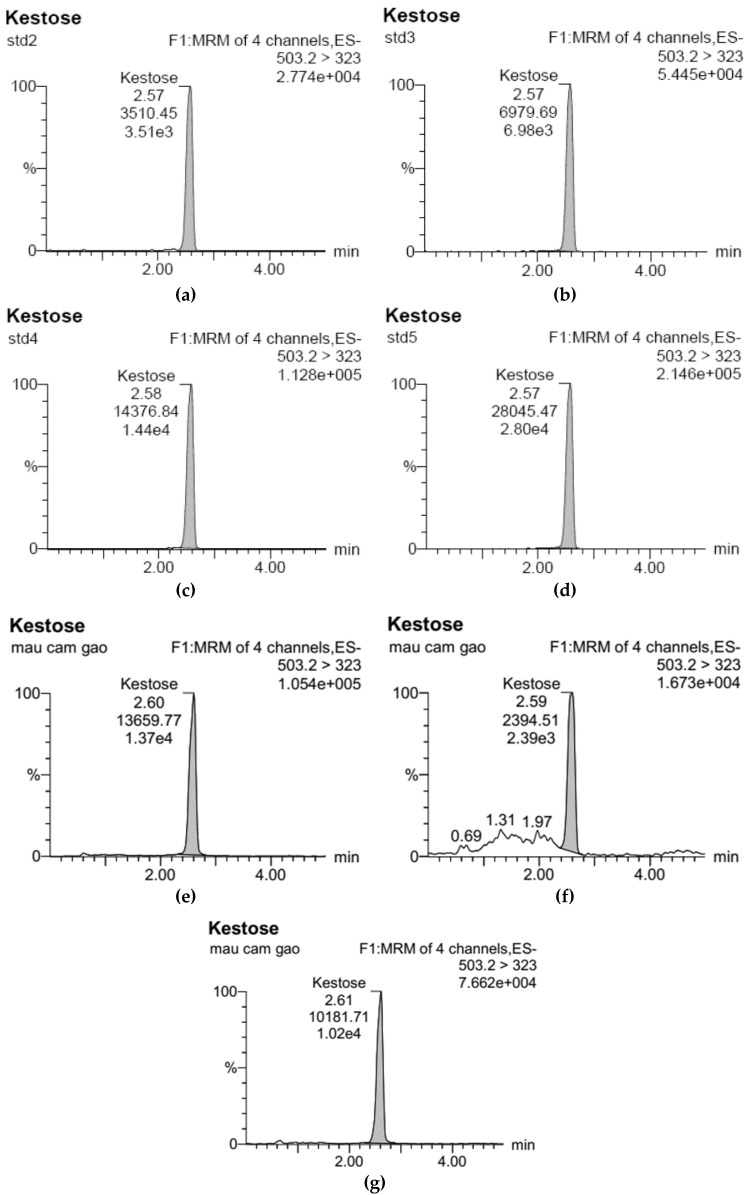
UPLC-ESI-MS/MS chromatograms for GF2 analysis: (**a**) Standard sample at concentration of 100 μg/L (std2); (**b**) of 200 μg/L (std3); (**c**) of 400 μg/L (std4); and (**d**) of 800 μg/L (std5); (**e**) Rice bran GF2 sample treated at pH 5.0 and 110 °C; (**f**) at pH 6.0 and 110 °C, and (**g**) at pH 7.0 and 110 °C; mau cam gao: rice bran.

**Figure 2:**
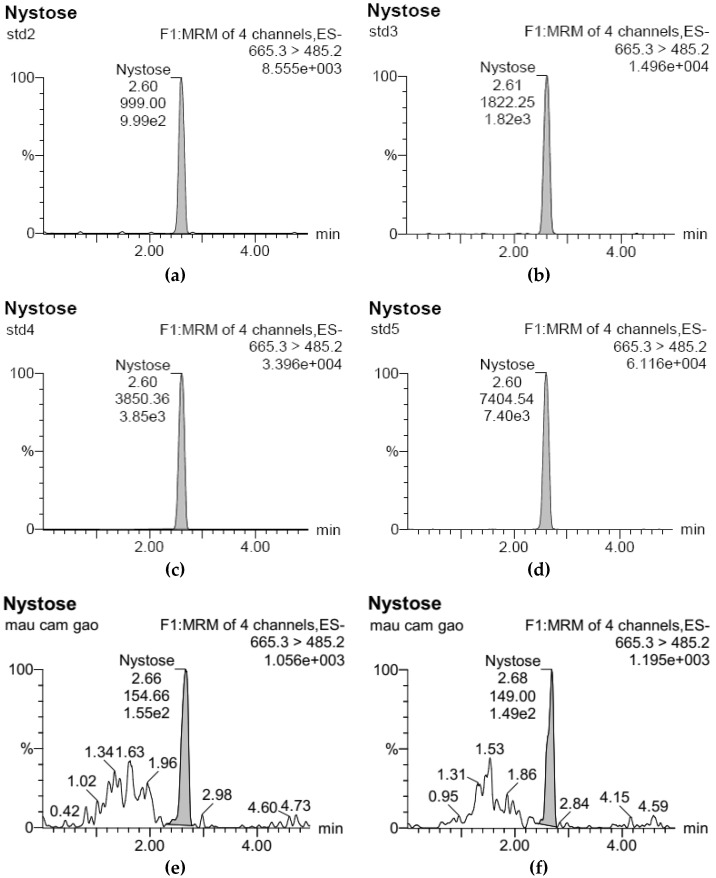

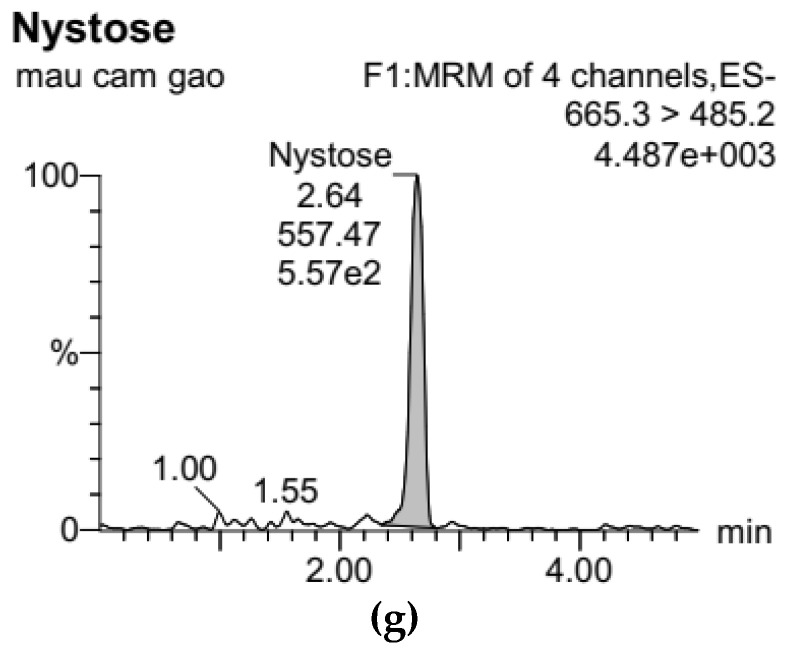
UPLC-ESI-MS/MS chromatograms for GF3 analysis: (**a**) Standard sample at concentration of 50 μg/L (std2); (**b**) of 100 μg/L (std3); (**c**) of 200 μg/L (std4); and (**d**) of 400 μg/L (std5); (**e**) Rice bran GF3 sample treated at pH 5.0 and 110 °C; (**f**) at pH 6.0 and 110 °C, and (**g**) at pH 7.0 and 110 °C; mau cam gao: rice bran.

**Figure 3:**
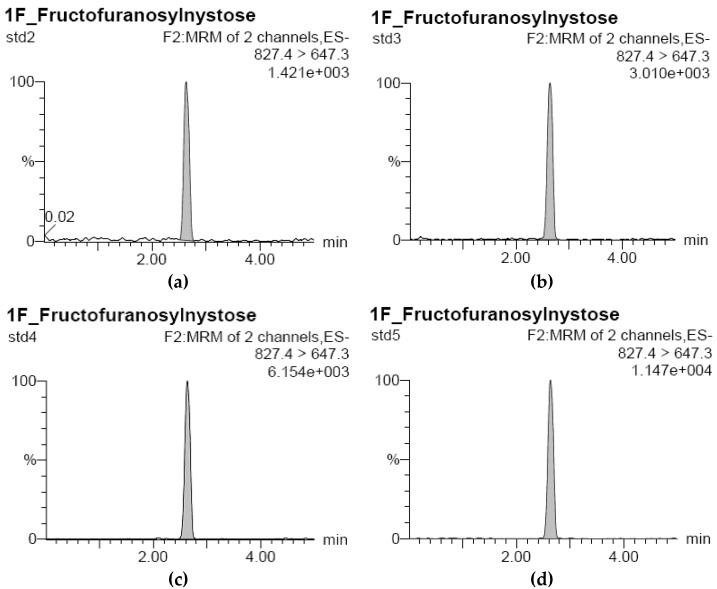

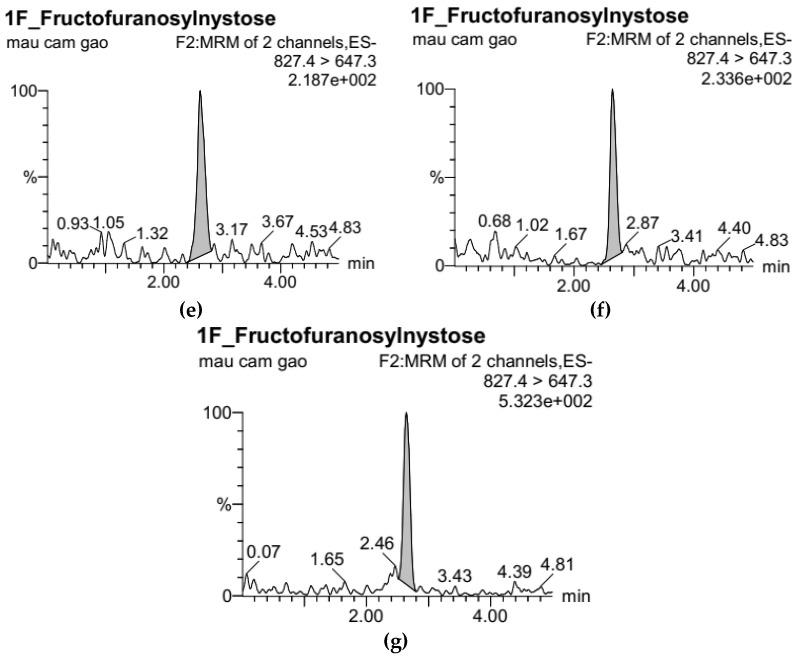
UPLC-ESI-MS/MS chromatograms for GF4 analysis: (**a**) Standard sample at concentration of 20 μg/L (std2); (**b**) of 40 μg/L (std3); (**c**) of 80 μg/L (std4); and (**d**) of 160 μg/L (std5); (**e**) Rice bran GF4 sample treated at pH 5.0 and 110 °C; (**f**) at pH 6.0 and 110 °C, and (**g**) at pH 7.0 and 110 °C; mau cam gao: rice bran.

The UPLC-ESI-MS/MS analysis of the standard FOS solutions (consisting of GF2, GF3, and GF4) was performed with analytical data presented in Table 2, Table 3 and Table 4, and the corresponding chromatograms for GF2, GF3, and GF4 are plotted in Figure 1, Figure 2 and Figure 3. Based on the data obtained, standard curves with good correlation coefficients (r^2^ equals 0.9997, 0.9992, and 0.9999 for GF2, GF3, and GF4, respectively) were constructed using regression analysis.

### 3.2. Thermal Degradation Kinetics of the Rice Bran FOS at Different pH Values

**Figure 4:**
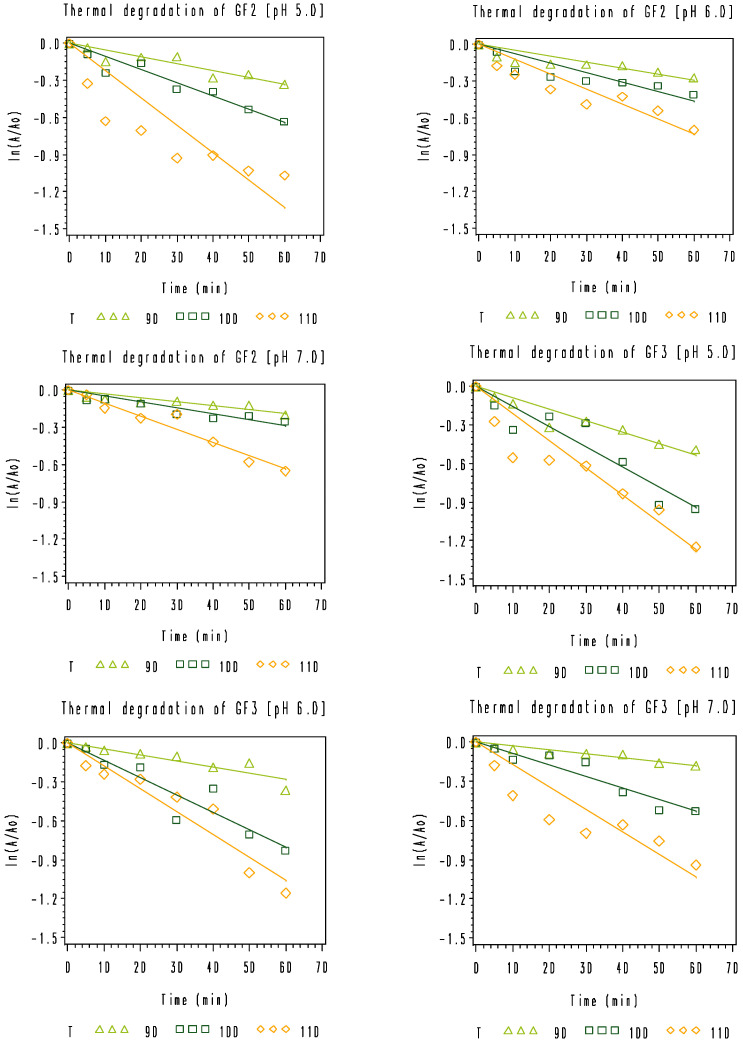

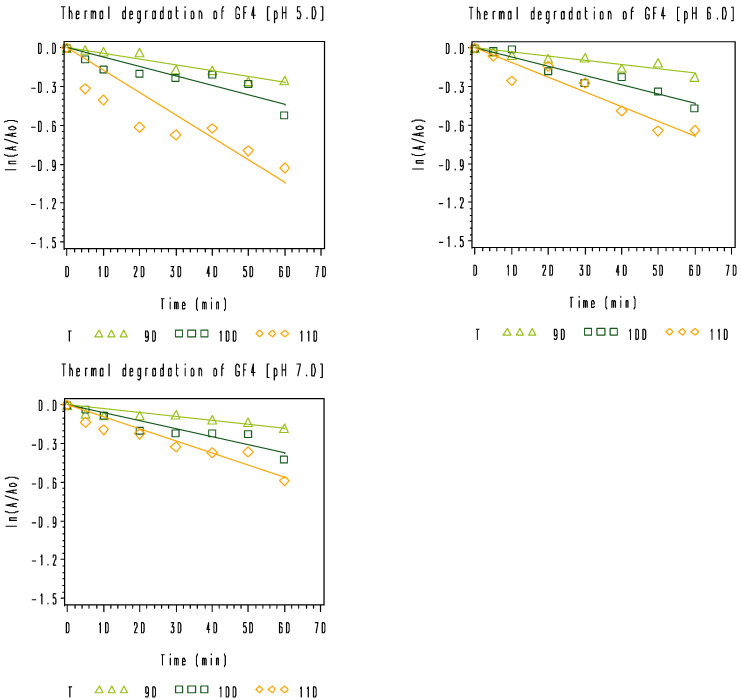
Thermal degradation of rice bran GF2, GF3, and GF4 dissolved in 0.2 M Na_2_HPO_4_/0.1 M citric acid buffers at pH 5.0, 6.0, and 7.0 at 90 °C (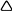), 100 °C (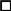), and 110 °C (◇).

**Figure 5:**
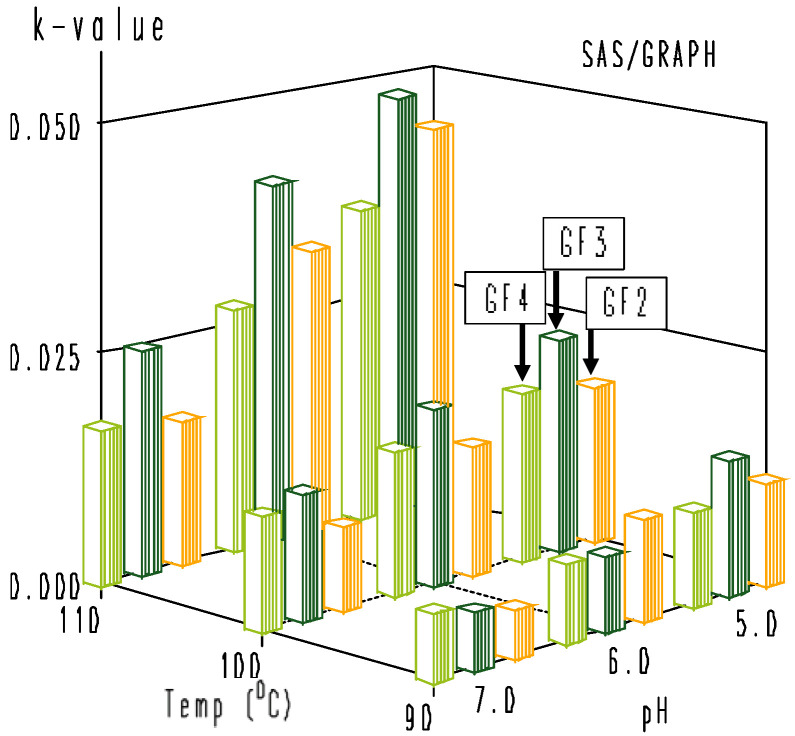
Estimated rate constants (min^−1^) of first-order degradation of rice bran GF2, GF3, and GF4 at different combinations of temperature and pH.

The effect of combined temperature and pH on the thermal degradation of rice bran-extracted GF2, GF3, and GF4 dissolved in buffered solutions was studied at 90, 100, and 110 °C and pH values of 5.0, 6.0, and 7.0. As observed, the combined temperature-pH degradation of the rice bran GF2, GF3, and GF4 samples could be adequately described by a first-order model (Equation (1)) in the temperature range of 90–110 °C (Figure 4). Degradation rate constants, *k* values, estimated using linear regression analysis of *ln(A*/*A*_0_*)* versus *t*, are reported in Table 5. As expected, the degradation rate constants increase with increasing temperatures at different pH values; however, the degradation rate constants decrease with increasing pH values. These findings are well in line with the data reported by L’homme et al. [2] for the study on pH-temperature hydrolysis of standard FOS dissolved in buffers, as those authors mentioned that the hydrolysis of standard FOS obeyed pseudo-first-order kinetics and took place much more easily at acidic pH than at neutral or basic pH values. As discussed and analytically proved by those authors [2], the stability of FOS is associated with the protonation of the breaking group. When the oxygen of the C-O osidic bond is protonated, the protonated oligosaccharides are more rapidly degradated at acidic pH than at neutral or basic pH values. Blecker et al. [27] reported that pseudo-first-order kinetics were found for the acid hydrolysis of five commercially available mixes of oligofructose samples. For a better view of the estimated rate constants of first-order degradation of rice bran GF2, GF3, and GF4 as a function of different combinations of temperature and pH, a three-dimension graph was constructed (Figure 5). As shown in Figure 5, the thermal degradation of rice bran-extracted GF2, GF3, and GF4 took place more easily at acidic pH than at neutral pH values. For each combination of temperature and pH, GF3 showed a faster degradation compared to GF2 and GF4. As mentioned by L’homme et al. [2], the concentration and water activity of FOS have an effect on their stability during treatments. In our experiments, the initial concentrations of GF2, GF3, and GF4 prepared from the rice bran crude extract were 669.32, 78.68, and 12.53 µg/L, respectively. The large difference in concentration of GF2, GF3, and GF4 might be one reason, among others, for the profound heat-pH sensitivity of GF3. On the other hand, the ionic strength of the buffers of pH 5.0 to 7.0 used for the dissolution of FOS during the experiments might interfere with the protonation of oxygen of the C-O osidic bond, leading to fast degradation of GF3.

**Figure 6:**
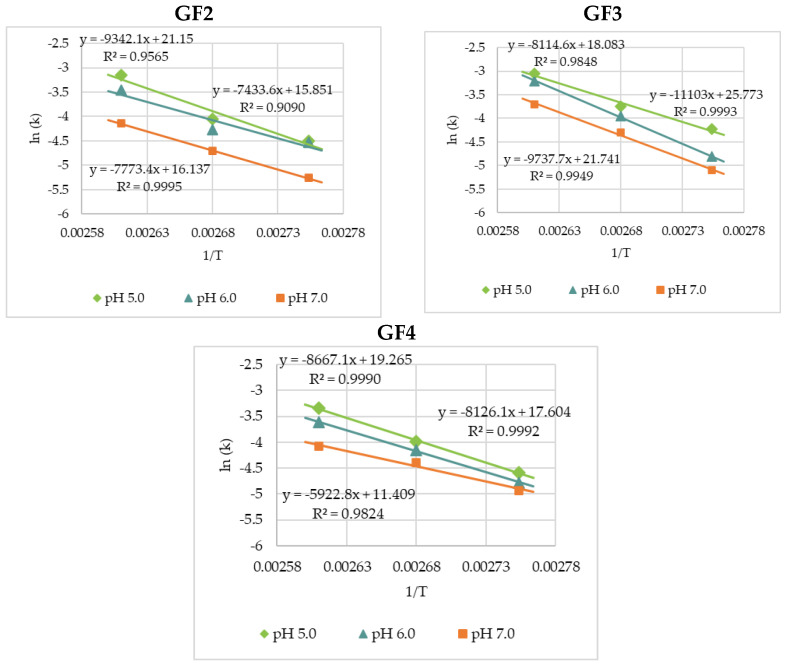
Heat dependence of *k*-values for the thermal degradation of rice bran GF2, GF3, and GF4 dissolved in 0.2 M Na_2_HPO_4_/0.1 M citric acid buffers at pH 5.0, 6.0, and 7.0.

As graphing *ln(k)* versus 1/*T* formed a linear line with a good correlation coefficient (Figure 6), the temperature dependence of the *k*-values, expressed in terms of activation energy (*E_a_*), in the temperature range studied, could be estimated using the Arrhenius equation (Equation (2)), with an activation energy range of 61.8–77.7 kJ·mol^−1^ obtained for GF2, 67.5–92.3 kJ·mol^−1^ for GF3, and 49.2–72.6 kJ·mol^−1^ for GF4 (Table 5). These findings are comparable with the data reported by L’homme et al. [2] for the study on pH-temperature hydrolysis of standard fructooligosaccharides dissolved in buffer at pH 7.0 with the activation energy ranging from 56.7 to 75.4 kJ·mol^−1^ for GF2, GF3, and GF4 being reported.

Table 6 shows the half-life time obtained for GF2, GF3, and GF4 dissolved in buffered solutions at pH 5.0, 6.0, and 7.0 through isothermal treatments at increasing temperatures from 90 to 110 °C. A 20-degree increase in the incubation temperature at pH 5.0 resulted in comparable decreases in the observed half-life values of GF2, GF3, and GF4 (3.9-fold, 3.2-fold, and 3.5-fold, respectively), while at pH 6.0 the decreases were 2.9-fold, 4.9-fold, and 3.2-fold, respectively, and at pH 7.0 those were 3.1-fold, 4.1-fold, and 2.3-fold, respectively.

### 3.3. Modeling of Combined Temperature and pH Dependence of Degradation Rate Constants

By fitting Equation (3) with *X*_1_ as the temperature variable and *X*_2_ as the pH variable on the experimental data, the model parameters were estimated using nonlinear regression analysis (proc NLIN, SAS). Based on the model parameters estimated, however, it was shown that the terms *β*_0_ and *β*_22_ were redundant, as indicated by the large standard error (~100%). As a consequence, these terms were omitted, and a reduced version of Equation (3) was used (i.e., Equation (4)). Model parameters estimated based on Equation (4) are shown in Table 7.

(4) $$Y=\beta_{1}X_{1}+\beta_{2}X_{2}+\beta_{11}X_{1}^{2}+\beta_{12}X_{1}X_{2}$$

**Figure 7:**
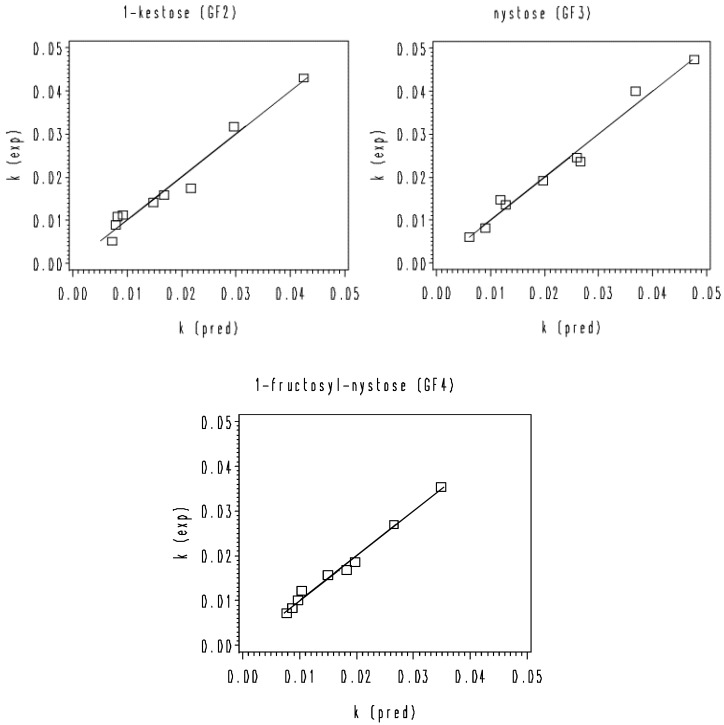
Correlation between the experimental *k* values and the estimated *k* values of the thermal degradation of rice bran GF2, GF3, and GF4 according to the second-degree polynomial model (Equation (4)).

For the re-constructed second-degree polynomial model (Equation (4)), no tendency was found by graphing residuals (differences between experimental and predicted *k* values, respectively) as a function of temperature, pH, experimental *k* value, and estimated *k* value (data not shown). In addition, the parity plots of the predicted *k* values based on Equation (4) versus the experimental *k* values were established for GF2, GF3, and GF4 (Figure 7). The deviation from the bisector can be considered an indicator of the inaccuracy of the models. The less the experimental and predicted *k* values mutually differ, the more successful the models are. Good agreements between the estimated *k* values and the experimental *k* values were observed for the aforementioned model version [31,35].

**Figure 8:**
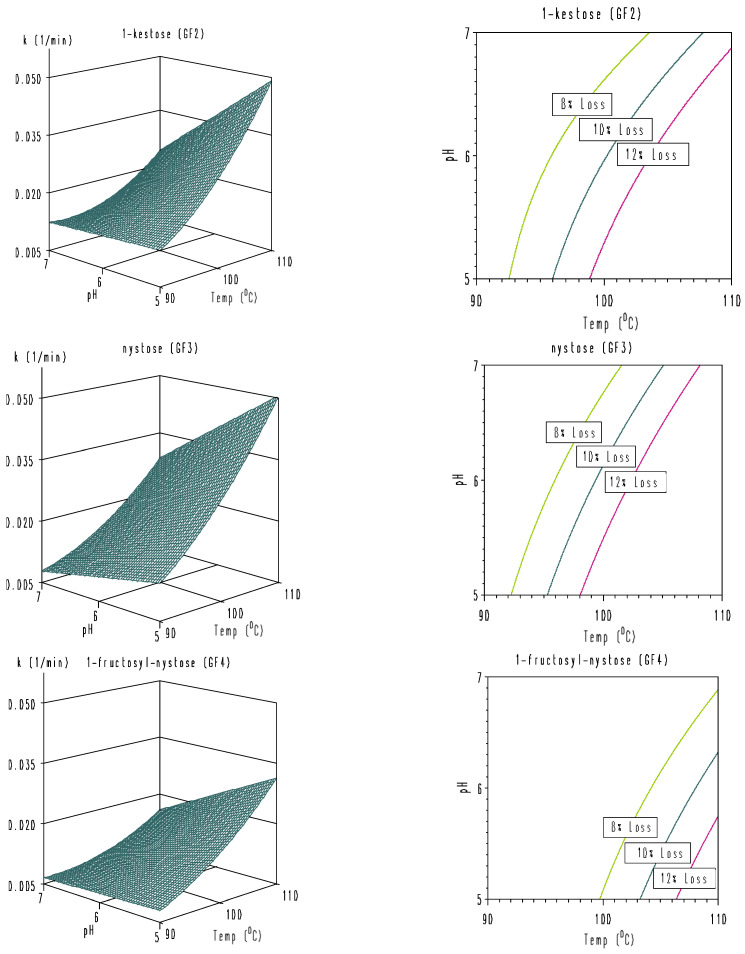
Three-dimensional and isorate contour plots for temperature-pH degradation of rice bran GF2, GF3, and GF4 dissolved in 0.2 M Na_2_HPO_4_/0.1 M citric acid buffers. Left: 3D plots; right: isorate contour plots for 8, 10, and 12% loss of GF2, GF3, and GF4 for a total process time of 5 min (*k* = 0.016676, 0.021072, and 0.025567 min^−1^, respectively) based on the second-degree polynomial model (Equation (4)).

By inserting all model parameters of Table 7 into Equation (4), heat-pH combinations resulting in specific preset degradation rate constants *k* of 0.016676, 0.021072, and 0.025567 min^−1^ corresponding to 8, 10, and 12% loss, respectively, of rice bran GF2, GF3, and GF4 for a total process time of 5 min were simulated and represented in isorate contour plots (Figure 8).

## 4. Conclusions

Rice bran-extracted GF2, GF3, and GF4 were rather thermally stable compounds at neutral pH while more labile at acidic pH. Among these, GF3 was more heat sensitive compared to GF2 and GF4. A mathematical equation was suggested for a description of the temperature-pH behavior of rice bran-extracted GF2, GF3, and GF4 during the processing of rice bran-based foods. This equation could be useful in designing alternative processing conditions for temperature-pH processing of rice bran-based products. Degradation kinetic studies of rice bran FOS in real food products would be interesting for food processors to evaluate the potential of temperature-pH processing of these products. Similar works can be applied for process stability studies of many other food quality attributes. This type of research is a good approach for the calculation and optimal design of processes for the food processing industry.

## Figures and Tables

**Table 1:** Program of mobile phase A and B for UPLC and fragmentation conditions for MS/MS measurement of GF2, GF3, and GF4.

No	Time (Min)	Flow Rate (mL/min)	% Acetonitril (Mobile Phase A)	% Water for UPLC (Mobile Phase B)
1	Initial	0.45	80	20
2	1.0	0.45	80	20
3	1.5	0.45	30	70
4	3.0	0.45	30	70
5	3.2	0.45	80	20
6	5.0	0.45	80	20
**Compound**	**Mode**	**Parent Ion (*m/z*)**	**Daughter Ion (*m/z*)**	**Cone Voltage (V)**
GF2	Negative	503.2	323.0	40
GF3	Negative	665.3	485.2	40
GF4	Negative	827.4	647.3	40

**Table 2:** Parameters for standard curve construction for GF2 analysis using UPLC-ESI-MS/MS.

Sample	Standard GF2 Concentration (μg/L)	RT (min)	Response	GF2 Concentration Based on Standard Curve (μg/L)
std1	0	0.00	0.00	0.00
std2	100	2.57	3510.45	98.16
std3	200	2.57	6979.69	196.99
std4	400	2.58	14,376.84	407.73
std5	800	2.57	28,045.47	797.12

**Table 3:** Parameters for standard curve construction for GF3 analysis using UPLC-ESI-MS/MS.

Sample	Standard GF3 Concentration (μg/L)	RT (min)	Response	GF3 Concentration Based on Standard Curve (μg/L)
std1	0	0.00	0.00	0.00
std2	50	2.60	999.00	50.78
std3	100	2.61	1822.25	95.44
std4	200	2.60	3850.36	205.48
std5	400	2.60	7404.54	398.30

**Table 4:** Parameters for standard curve construction for GF4 analysis using UPLC-ESI-MS/MS.

Sample	Standard GF4 Concentration (μg/L)	RT (min)	Response	GF4 Concentration Based on Standard Curve (μg/L)
std1	0	0.00	0.00	0.00
std2	20	2.63	160.80	19.46
std3	40	2.64	351.50	40.93
std4	80	2.63	694.50	79.54
std5	160	2.63	1409.80	160.06

**Table 5:** Estimated rate constants, *k* values (min^−1^), of first-order degradation of rice bran GF2, GF3, and GF4 (in 0.2 M Na_2_HPO_4_/0.1 M citric acid buffers at pH 5.0, 6.0, and 7.0) at different combinations of temperature and pH.

	pH	90 °C	100 °C	110 °C	*Ea* (kJ·mol^−1^)	R^2^
GF2	5.0	0.0112 ± 0.0012 ^a^	0.0174 ± 0.0020	0.0430 ± 0.0056	77.7	0.9565
	6.0	0.0109 ± 0.0011	0.0141 ± 0.0015	0.0318 ± 0.0031	61.8	0.9090
	7.0	0.0052 ± 0.0005	0.0090 ± 0.0008	0.0159 ± 0.0012	64.6	0.9995
GF3	5.0	0.0147 ± 0.0011	0.0236 ± 0.0020	0.0473 ± 0.0052	67.5	0.9848
	6.0	0.0081 ± 0.0009	0.0192 ± 0.0016	0.0401 ± 0.0030	92.3	0.9993
	7.0	0.0061 ± 0.0005	0.0136 ± 0.0014	0.0246 ± 0.0026	81.0	0.9949
GF4	5.0	0.0102 ± 0.0009	0.0186 ± 0.0020	0.0353 ± 0.0041	72.1	0.9990
	6.0	0.0084 ± 0.0006	0.0157 ± 0.0011	0.0270 ± 0.0031	67.6	0.9992
	7.0	0.0072 ± 0.0007	0.0123 ± 0.0011	0.0169 ± 0.0014	49.2	0.9824

^a^ Standard error of regression.

**Table 6:** Estimated half-life time (min) of rice bran GF2, GF3, and GF4 (in 0.2 M Na_2_HPO_4_/0.1 M citric acid buffers at pH 5.0, 6.0, and 7.0) at different combinations of temperature and pH.

	pH	90 °C	100 °C	110 °C
GF2	5.0	62.1	39.8	16.1
	6.0	63.9	49.3	21.8
	7.0	133.7	76.9	43.7
GF3	5.0	47.2	29.4	14.7
	6.0	85.2	36.2	17.3
	7.0	113.9	51.0	28.1
GF4	5.0	68.3	37.3	19.6
	6.0	82.7	44.1	25.7
	7.0	96.2	56.4	41.1

**Table 7:** Estimated model parameters for temperature-pH degradation of rice bran GF2, GF3, and GF4 based on the second-degree polynomial model (Equation (4)).

Parameter	GF2	GF3	GF4
*β*_1_ *(X*_1_ *: temp)*	−0.00349 ± 0.00083	−0.00256 ± 0.00072	−0.00222 ± 0.00037
*β*_2_ *(X*_2_ *: pH)*	0.0523 ± 0.0133	0.0330 ± 0.0115	0.0318 ± 0.0059
*β*_11_ *(X*_1_^2^ *: temp* ^2^ *)*	0.000041 ± 0.000008	0.000032 ± 0.000007	0.000026 ± 0.000003
*β*_12_*(X*_1_ * X_2_: temp * pH)	−0.00059 ± 0.00013	−0.00040 ± 0.00012	−0.00036 ± 0.00006
*Corrected R* ^2^	0.984	0.992	0.995
*Standard Deviation (SD)*	0.0029	0.0025	0.0013

## Data Availability

The data are contained within the article.
